# ENPP1 inhibition as a therapeutic approach for later-onset hypophosphatasia

**DOI:** 10.1093/jbmr/zjaf136

**Published:** 2025-10-06

**Authors:** Sonoko Narisawa, Flavia Amadeu de Oliveira, Cintia Kazuko Tokuhara, Elis J Lira dos Santos, Elena Fonfria, Jennifer Batson, Zhiliang Cheng, Ann Houston, Brian L Foster, José Luis Millán

**Affiliations:** Sanford Children's Health Research Center, Sanford Burnham Prebys Medical Discovery Institute, La Jolla, CA, 92037, United States; Sanford Children's Health Research Center, Sanford Burnham Prebys Medical Discovery Institute, La Jolla, CA, 92037, United States; Sanford Children's Health Research Center, Sanford Burnham Prebys Medical Discovery Institute, La Jolla, CA, 92037, United States; Division of Biosciences, College of Dentistry, The Ohio State University, Columbus, OH 43210, United States; Recursion, Salt Lake City, UT 84101, United States; Recursion, Salt Lake City, UT 84101, United States; RallyBio, New Haven, CT 06510, United States; RallyBio, New Haven, CT 06510, United States; Division of Biosciences, College of Dentistry, The Ohio State University, Columbus, OH 43210, United States; Sanford Children's Health Research Center, Sanford Burnham Prebys Medical Discovery Institute, La Jolla, CA, 92037, United States

## Abstract

Hypophosphatasia (HPP) is caused by loss-of-function mutations in the human *ALPL* gene that encodes tissue-nonspecific alkaline phosphatase (TNAP), whose deficiency results in the accumulation of the calcification inhibitor inorganic pyrophosphate (PP_i_), resulting in skeletal and dental hypomineralization. Enzyme replacement with mineral-targeted TNAP (asfotase alfa) improves skeletal mineralization but the almost daily injections of this biologic can lead to injection site reactions and discontinuation of treatment. Since PP_i_ is produced by the enzymatic action of ectonucleotide pyrophosphatase/phosphodiesterase 1 (ENPP1) from adenosine triphosphate (ATP), we tested if ENPP1 could be a druggable target for the development of an alternative treatment for HPP, particularly for the non-lethal later-onset forms of HPP, where enzyme replacement is not currently approved. We orally administered 30 and 100 mg/kg/day of an ENPP1 inhibitor, REV102, to the *Alpl^Prx1/−^* mouse model of late-onset HPP, for 105 days and confirmed target engagement, as plasma PP_i_ concentrations were markedly reduced. X-ray, micro-CT, and bone morphometry indicated improvement in appendicular skeletal mineralization. This study suggests that the adult HPP phenotype could benefit from oral administration of ENPP1 inhibitors.

## Graphical Abstract

**Graphical Abstract:**
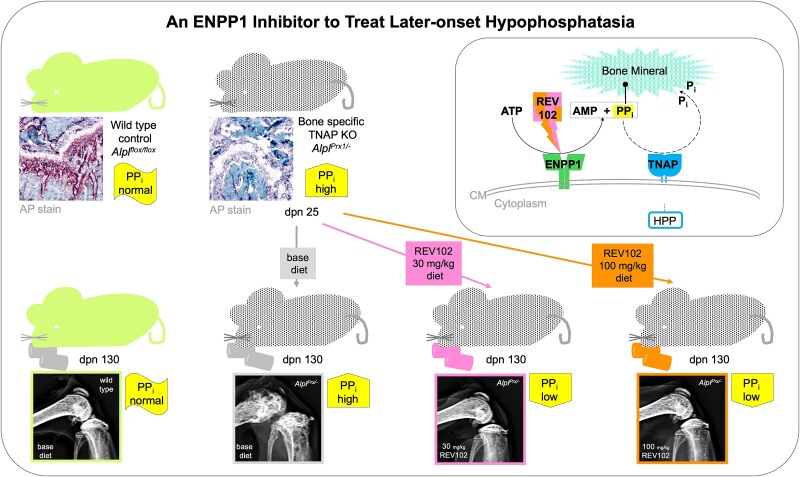

## Introduction

Extracellular inorganic pyrophosphate (PP_i_) is a potent inhibitor of mineralization^1^ and is considered the body’s natural water softener^2^ as it efficiently prevents inappropriate soft tissue calcification. In blood, PP_i_ concentrations are in the μM range while phosphate (P_i_) levels are in the mM range and as a result, small changes in the PP_i_/P_i_ ratio have profound implications for the control of soft-tissue calcification. The major producer of inorganic PP_i_ is ectonucleotide pyrophosphatase/phosphodiesterase 1 (ENPP1), and enzyme which hydrolyzes ATP to AMP and PP_i_. ENPP1 deficiency leads to ossification of the posterior longitudinal ligament of the spine (OPLL, Online Mendelian Inheritance of Man [OMIM] 602475), generalized arterial calcification of infancy (GACI, OMIM 208000), but can also manifest phenotypic changes characteristic of pseudoxanthoma elasticum (PXE, OMIM 264800), that is caused by a deficiency in the ABCC6 transporter. Enpp1^−/−^ mice have almost undetectable levels of plasma PP_i_ leading to inappropriate deposition of hydroxyapatite in soft tissues.^3^^,^^4^ Interestingly, *Enpp1*^−/−^ mice also display reduced trabecular and cortical bone in the long bones and decreased bone strength^5^ phenocopying autosomal recessive hypophosphatemic rickets type 2 in humans (ARHR2, OMIM 613312)^6^ and those children that survive GACI (experiencing the characteristic calcification of their arteries, heart, kidneys, and joints) will develop ARHR2 (displaying rickets, bone and muscle pain, bowing of the legs, short stature, and an increased risk of fractures).

The major enzyme controlling the all-important PP_i_/P_i_ ratio is tissue-nonspecific alkaline phosphatase (TNAP), which generates P_i_ locally from extracellular ATP, while its pyrophosphatase (PP_i_ase) activity is crucial in controlling extracellular PP_i_ concentrations, as clearly evidenced by disorders where TNAP expression is either reduced or enhanced. Hypophosphatasia (HPP) is caused by loss-of-function mutations in the human *ALPL* gene that encodes TNAP, whose deficiency results in the accumulation of PP_i_.^7^ Skeletal and dental hypomineralization characterizes HPP, with disease severity varying from life-threatening perinatal or infantile forms (OMIM 251500), to childhood (OMIM 251510), or to the milder forms that manifest in adulthood or only affect the dentition (OMIM 146300).^8^ In contrast, overexpressing TNAP in the muscular medial layer of arteries leads to medial artery calcification,^9^ a serious clinical condition that develops in patients with diabetes, obesity, chronic kidney disease (CKD)-mineral bone disorder, and simply during aging, while overexpression of TNAP in the intimal layer of arteries is a key event in the pathophysiology of atherosclerosis.^10^^,^^11^

We previously conducted pre-clinical studies that led to the development of a novel enzyme replacement therapy using a recombinant mineral-targeted form of TNAP (sALP-Fc-D_10_, aka asfotase alfa),^12–16^ which was approved in 2015 for the treatment of pediatric-onset HPP. It markedly improves lifespan, the skeletal phenotype, motor function, and the quality of life of patients with HPP.^17^ However, enzyme replacement necessitates 3-6 injections of the biologic per week, often leading to injection site reactions severe enough to force discontinuation of treatment.^18^^,^^19^ Just recently, we achieved a considerable improvement of this therapeutic principle using a single intramuscular administration of adeno-associated virus 8 encoding TNAP-D_10_ to increase the lifespan and improve the skeletal and dentoalveolar phenotypes in both the *Alpl^−/−^* model of severe infantile HPP^20–22^ and the *Alpl^Prx1/Prx1^* model of later-onset HPP,^22^^,^^23^ obviating the need for the multiple weekly injections. This viral vector therapy is awaiting initiation of clinical trials.

Earlier genetic studies by our group demonstrated that the double ablation of the *Alpl* and *Enpp1* genes (*Alpl^−/−^; Enpp1^−/−^* double knockout mice) led to normalization of plasma PP_i_ concentrations and improvements in skeletal mineralization^4^^,^^5^ suggesting that both ENPP1 and TNAP are potentially useful druggable targets for diseases caused by dysregulated PP_i_ metabolism. Indeed, upregulation of TNAP in soft tissues leads to sinking PP_i_ levels and wide-spread ectopic calcification^24^ and TNAP inhibition using SBI-425^25^^,^^26^ is able to reduce ectopic calcification in animal models of PXE,^27^^,^^28^ CKD,^29^ and atherosclerosis.^30^ Here, we tested the potential efficacy of using ENPP1 inhibition as a means of sinking PP_i_ concentrations in an *Alpl^Prx1/−^* mouse model of later-onset HPP.^31^ We used the ENPP1 inhibitor REV102 (RallyBio and Recursion) and administered this small molecule admixed to the mouse chow for 105 d followed by X-ray, micro-CT, and bone morphometry analyses. Our data indicate that the adult HPP phenotype could benefit from oral administration of an ENPP1 inhibitor.

## Materials and methods

### Animal model and dosing

*Alpl^flox/flox^* (*Alpl^tm3.1Jlm^*) mice were crossed to a *Prx1-Cre* transgenic mouse (B6.Cg-Tg(Prrx1-cre)1Cjt/J) to generate *Prx1-Cre; Alpl^flox/flox^* (*Alpl^Prx1/Prx1^*).^31^ Among the offspring animals, we identified *Alpl^Prx1/−^* mice that carry one allele of *Alpl* gene conditionally inactivated and the second *Alpl* allele constitutively deleted, since the *Prx1-Cre* gene is often expressed in the germ cells, producing offspring with a constitutively inactivated *Alpl* allele. The bone phenotype of *Alpl^Prx1/−^* is slightly more pronounced than that of *Alpl^Prx1/Prx1^* mice, as their plasma ALP levels are approximately 50% of the *Alpl^Prx1/Prx1^*. Thus, we used *Alpl^Prx1/−^* mice as a model of later-onset HPP in this study.

Breeding pairs of [*Prx1-Cre; Alpl^flox/−^*] × *Alpl^flox/flox^*, [*Prx1-Cre; Alpl^flox/flox^*] × *Alpl^flox/−^*, [*Prx1-Cre; Alpl^flox/flox^*] × *Alpl^flox/flox^*, and the reverse of dam × sire pairs in these three combinations were set up, and offspring pups were genotyped by PCR using DNA extracted from toe cut biopsies. The three primers, 5′-TTG CCT GGA ACC TGT TCA CA-3′, 5′-CCA GTC CAT GTG CAG CTA CA-3′, and 5′-GAT GAC ATT CTT GGC TAC ATT GGT-3′ were used to amplify 283 bp wild-type, 317 bp floxed, and 415 bp Cre’d fragments. The Prx1-Cre transgene was identified with two primers, 5′-ATG GTG TTG CCG CGC CAT CTG CCA-3′ and 5′-CTA ATC GCC ATC TTC CAG CAG GCG C-3′. Toe cuts were also used as mouse ID numbers in each litter. Ear notches were also given as identification (ID) at the time of weaning (postnatal days 17-20). Mice were assigned to each group randomly by a researcher who had not seen the actual animals, while original littermates were often placed in a single cage with the same diet. The numbers of mice per cage were 1-4. We placed an enrichment item (Mouse Igloo) to all the singly housed animals.

Pellet diets (#2918, Teklad Global 18% Protein-6% fat Rodent Diet, irradiated) containing REV102 at concentrations of 0, 0.188, and 0.625 g/kg were used for dosing the HPP mice, corresponding to 0, 30, and 100 mg/kg body weight under the assumptions that each mouse weighs approximately 25 g and consumes 4 g pellet diet per day. The three diets were prepared by Inotiv (https://www.inotiv.com). The diet was given ad libitum and renewed once a week, starting at postnatal day (dpn) 25 until collection day, at dpn 130. Body weight was monitored every 20th day. Wild-type control mice (*Alpl^flox/flox^*) were given the base diet, #2918, containing 0 mg/kg REV102 and collected on dpn 130.

Mice were anesthetized with Avertin (0.017 mL/g body weight). Blood was collected by cardiac puncture and transferred to a lithium heparin tube (BD365987) and an EDTA tube (Sarstedt 20.1341.102). Heparin plasma after immediate filtration with Spin-X column (Coster 8160) and EDTA plasma were quickly frozen down on dry ice and stored at −80 °C until analyses. For certain female mice, after removing muscle tissue, femur samples were stored in RNAlater, along with liver and kidney, for RNA analysis. Tibia bones without muscle tissue, one-half of a kidney, and part of the liver were directly frozen down on dry ice and stored at −80 °C for protein analysis. Skeletal samples were fixed in 4% PFA/PBS and stored at 4 °C for analyses, including X-rays, micro-CT, and bone histomorphometry.

All animal procedures in this study were approved by the Institutional Animal Care and Use Committee at Sanford Burnham Prebys Medical Discovery Institute (AUF #21-017, 24-033), complying with Animal Welfare Act Regulations and Guide for the Care and Use of Laboratory Animal. The mice were maintained according to animal facility breeding standard operating procedures (SOPs). Animal holding rooms provide a specific pathogen-free environment, 12-h light/12-h dark cycle, approximately 25 °C room temperature and 45%-55% humidity.

### ENPP1 inhibitor

REV102 (aka REC102) was synthesized in-house and kindly supplied by RallyBio-Recursion. REV102 is a small molecule reversible inhibitor of ENPP1. The ability of REV102 to inhibit the activity of mouse ENPP1 was assessed using an adenosine monophosphate/guanosine monophosphate (AMP/GMP) time-resolved energy fluorescence (TR-FRET) assay. REV102 inhibited mouse ENPP1 enzymatic activity with an IC_50_ of 6.3 nM. Briefly, ENPP1 catalyzes the hydrolysis of ATP into AMP+PP_i_. AMP production was monitored using Bellbrooks Transcreener AMP/GMP TR-FRET assay kit (Cat No. 3020-10K), using 15 pM mouse ENPP1 protein (BCS Cat No. 221201-LZY221216-P1) in the presence of 400 nM ATP. AMP produced in the assay by ENPP1 activity competes with a far-red tracer labeled AMP bound to an AMP specific antibody resulting in a decrease in TR-FRET. ENPP1 inhibition reduces AMP production and increases the TR-FRET signal.

### Plasma analysis

Heparin plasma was deproteinized with Nanosep 10kD cutoff column (Pall OD010C34) and PP_i_ levels were measured following a standard procedure using ATP Sulfurylase (R&D, 7175-AS-020).^20^ Alkaline phosphatase (AP) activity was measured using pNPP as a substrate and a recombinant human TNAP as a standard.^32^ Plasma levels of osteopontin (OPN) were assessed by Quantine ELISA (MOST00, R&D) by following the manufacturer’s protocol. All these measurements, utilized in 96-well microtiter plates, were processed by Spectra Max iD3 (Molecular Devices). To evaluate the health condition after 105-d dosing, heparin plasma was analyzed by using Comprehensive Diagnostic Profile rotors for the VetScan VS2 analyzer (Abaxis). EDTA plasma was used for the measurement of REV102 levels by a standard HPLC analysis operated by Pharmaron Lab Services LLC.

### Imaging analysis

X-ray views were obtained using the Trident Specimen Radiography System (Hologic) at an energy of 25 kV. Images were saved as TIFF files using MicroDICOM Viewer software.

For micro-CT, fixed samples were scanned in a micro-CT (Scanco Medical), at 70 kV, 76 μA, 0.5 mm Al filter, 900 ms integration time, and 10 μm voxel dimension. A set of five hydroxyapatite phantoms of known density was used to calibrate the scans. Reconstructed DICOM files were exported and analyzed using Analyze version 15.0 (AnalyzeDirect) as previously described.^22^ Trabecular and cortical bone of femurs were segmented at 400 and 550 mg hydroxyapatite (HA)/cm^3^, respectively. Micro-CT analysis of femurs was performed as previously described. Briefly, to quantify the trabecular bone parameters, such as bone volume (Tb.BV), total volume (Tb.TV), bone volume fraction (Tb.BV/TV), trabecular number (Tb.N), thickness (Tb.Th), spacing (Tb.Sp), connective density (Tb.Conn.D), and mineral density (Tb.BMD), a total of 0.5 mm (50 slices) proximal to the distal femur growth plate was traced. For the cortical bone, 50 slices of the mid-femur of each bone were used to quantify cortical bone volume fraction (Ct.BV/TV), cortical thickness (Ct.Th), marrow area (Ma.Ar), and mineral density (Ct.BMD).

### Bone morphometry and histology

Femur and spine sections were prepared as before,^32^ except that we used bone samples fixed and stored in 4% PFA. Briefly, fixed femur and spine samples were rapidly frozen down in SCEM embedding media using Hexanes dry-ice bath. Sections (approximately 5 μm) were obtained with a tungsten blade set up in a −18 °C Cryostat (Leica 1850). Sections adhered to the Kawamoto film were defrosted and air-dried for 10 min before re-fixation in 4% PFA for 60 min. Rinsed sections were processed for a standard Von Kossa staining method using silver nitrate solution and Nuclear Fast Red for the counterstain. Pictures were taken with Keyence microscope BZ-X800 and the mineralized area was measured by using a function of Bright Field-Single Extraction in the BZ-X800 Analyzer. Distal femur sections prepared by the Kawamoto method were also stained with a standard protocol of Goldner’s trichrome stain.

### Quantitative reverse transcription polymerase chain reaction (qRT-PCR)

RNA was extracted from liver and kidney using the RNeasy Plus kit (Qiagen) following the manufacturer’s protocol. Femur bone was first frozen down in liquid nitrogen and ground into powder using a pre-cooled pestle and a mortar placed over liquid nitrogen. The bone powder was first extracted with Solution D and phenol-chloroform and RNA fraction obtained was re-purified using RNeasy Plus kit. About 2 μg of total RNA was reverse transcribed with qScript cDNA SuperMix (QuantaBio), and PCR reactions were prepared with qPCR Master Mix (BioPioneer). Quadruplet samples were amplified by 7900 HT (Applied Biosystems). Sequences of the primers are shown in the Table S1.

### Western blots

Protein extracts were prepared in radioimmunoprecipitation assay (RIPA) buffer by standard procedure except that frozen tibia samples were first ground into bone powder prior to homogenization in RIPA buffer. Protein concentration was determined by bicinchoninic acid assay reagent (Pierce, Thermo Fisher) and 100 μg protein was loaded in each lane of 10% acrylamide gel (Novex/Thermo Fisher) followed by a standard immunoblot protocol. Antibodies used were: ENPP1/NPP1 (Invitrogen, PA5-18429), P_i_T1 (Santa Cruz, sc-98 814), CD73 (Cell Signalling13160), and OPN (Abcam, AB283656). A recombinant mouse OPN (Sigma) was used as a positive control. Chemiluminescence signals with ECL Prime Western Blotting Detection Reagents (Cytiva) were analyzed using ChemiDoc Imaging systems (Bio-Rad).

All the graphs were prepared with Prism 10 software (GraphPad), and two groups were compared using the Student’s *t*-test. When differences were statistically significant (*p* < .05), graphs were labeled with * marks.

## Results

### ENPP1 inhibitor-treated *Alpl*^*Prx1/−*^ mice grew normally and showed markedly reduced plasma PP_i_

**Figure 1:**
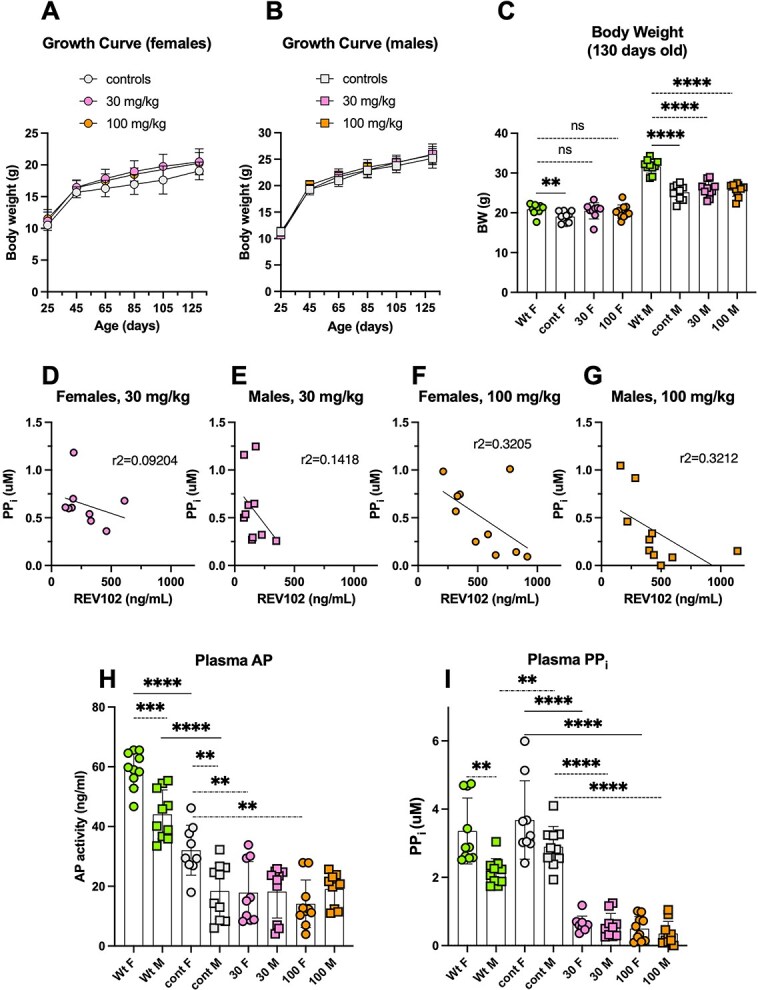
Growth curves of female (A) and male (B) mice during the 105 d dosing with REV102. Body weight comparison at 130 dpn. (C). Correlation of REV102 drug levels and PP_i_ in the terminally collected plasma from females fed a 30 mg/kg dose (D), males fed a 30 mg/kg dose (E), females fed a 100 mg/kg dose (F), and males fed a 100 mg/kg dose (G). Alkaline phosphatase (AP) levels in the plasma at dpn 130 (H). PP_i_ levels in the plasma at dpn 130 (I). F: female, M: male, Wt: wild-type (*Alpl^flox/flox^*) fed a base diet (female and male, *n* = 10), control: *Alpl^Prx1/−^* mice fed a base diet (female *n* = 9, male *n* = 10), 30: *Alpl^Prx1/−^* mice fed det supplemental with a 30 mg/kg REV102 dose (female *n* = 9, male *n* = 10), 100: *Alpl^Prx1/−^* mice fed a diet supplemented with a 100 mg/kg REV102 dose (female and male *n* = 10).

Female and male *Alpl^Prx1/−^* mice who consumed test diets containing REV102 from 25 dpn grew normally and gained weight comparably to control untreated *Alpl^Prx1/−^* mice (Figure 1A and B). Body weight at collection time (130 dpn) showed that dosed females gained weight to the levels of wild-type (Wt) females, while dosed males did not reach the weight levels of age-matched Wt controls (Figure 1C). In the circulation of dosed animals, REV102 levels negatively correlated with PP_i_ concentrations (Figure 1D-G). Female and male mice dosed with either 30 or 100 mg/kg tended to show decreased PP_i_ levels in plasma (Figure 1F and G).

Plasma AP levels were higher in female Wt mice than in Wt males (Figure 1H, *p* = .0002). Control female mice also showed higher AP levels compared to control male mice (Figure 1H, *p* = .0038). Dosed female *Alpl^Prx1/−^* mice show lower values than control female mice (cont F vs 30 F *p* = .0056, cont F vs 100 F *p* = .0002), while plasma AP levels in male mice, both dosed *Alpl^Prx1/−^* and control *Alpl^Prx1/−^* mice, did not show a difference.

Figure 1D-G shows plasma PP_i_ levels at collection time (dpn 130). PP_i_ levels in control *Alpl^Prx1/−^* mice are slightly elevated compared to Wt control mice (females: mean 3.682 mM vs 3.359 mM, *p* = .5144, males: mean 2.897 mM vs 2.161 mM, *p* = .0044). Interestingly, while Wt female mice had higher PP_i_ levels than Wt male mice (*p* = .0019), sex differences were not found among *Alpl^Prx1/−^* mice. With the 30 mg/kg dose, PP_i_ concentrations were reduced to approximately 17.3% and 20.2% of the untreated control mice, female or male mice, respectively. With the 100 mg/kg dose, PP_i_ concentrations were reduced to approximately 13.5% and 12.2% of the control *Alpl^Prx1/−^* mice, female and male mice, respectively. The PP_i_ levels in 100 mg/kg dose appear slightly lower than 30 mg/kg dose; however, the differences were not statistically significant in either female or male mice.

### Improved bone phenotype in *Alpl*^*Prx1/−*^ mice dosed with the ENPP1 inhibitor

**Figure 2:**
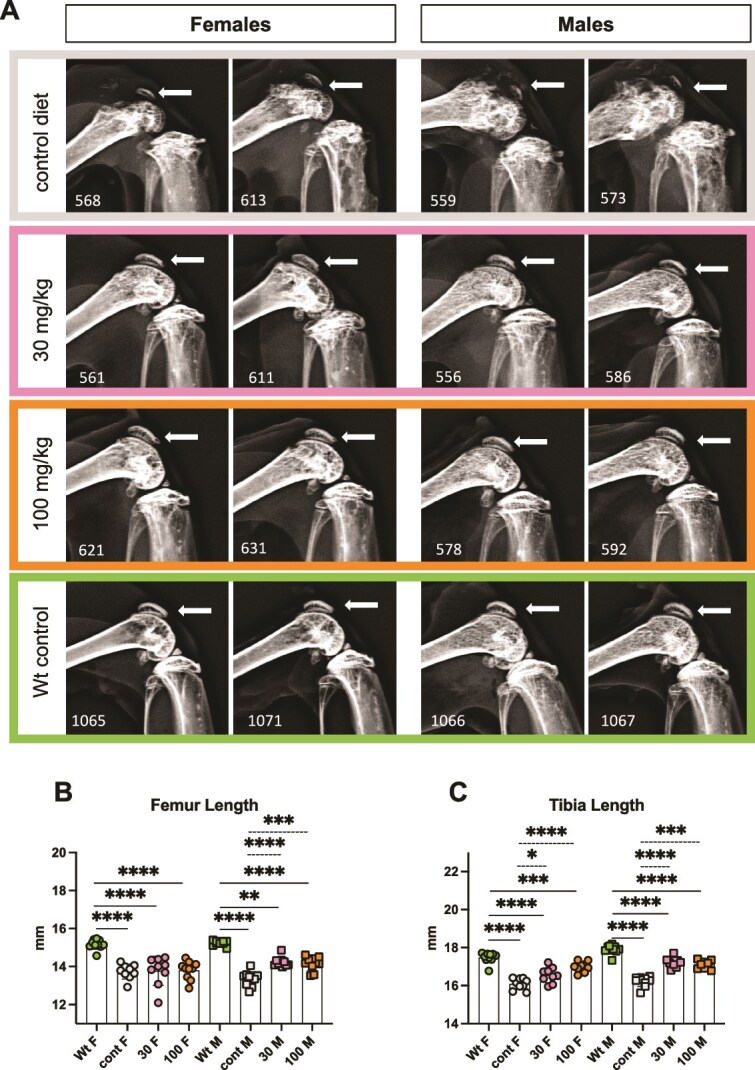
Representative X-ray images of the left hind limb joints (A). Images were saved with automatic exposure in the Trident X-ray device, and no modifications were done. Numbers at the bottom left corner in each picture are mouse ID numbers. The *Alpl^Prx1/−^* mice show diminished patella structure but all the treated mice present patella indistinguishable from Wt controls (white arrows). Wt: wild-type mice *Alpl^flox/flox^*, cont: *Alpl^Prx1/−^*, 30: *Alpl^Prx1/−^* mice with 30 mg/kg REV102, 100: *Alpl^Prx1/−^* mice with 100 mg/kg. Comparison of length of femur (B) and tibia (C) obtained using ImageJ software from the X-ray images. REV102. (B) Cont M vs 30 M *p* < .0001, cont M vs 100 M *p* = .0001. (C) Cont F vs 30 F *p* = .0139, cont F vs 100 F *p* < .0001, cont M vs 30 M *p* < .0001, cont M vs 100 M *p* < .0001.

Both male and female untreated *Alpl^Prx1/−^* mice present poorly mineralized, widened femoral epiphysis with highly abnormal patellas (arrows in Figure 2A, top row), but all the treated *Alpl^Prx1/−^* animals showed improved appearance of the epiphysis with a well-defined patella structure, undistinguishable from Wt control mice by X-ray imaging (Figure 2A and Figures S2 and S3). The length of the femurs in female *Alpl^Prx1/−^* mice was not changed by REV102 treatment, but the length of the femurs of treated *Alpl^Prx1/−^* male mice increased (Figure 2B). The length of the tibia increased by the treatment in both male and female *Alpl^Prx1/−^* mice (Figure 2C). Vertebrate/spine samples from *Alpl^Prx1/−^* mice were not distinguishable from Wt mice in X-ray images (Figure S4).

**Figure 3:**
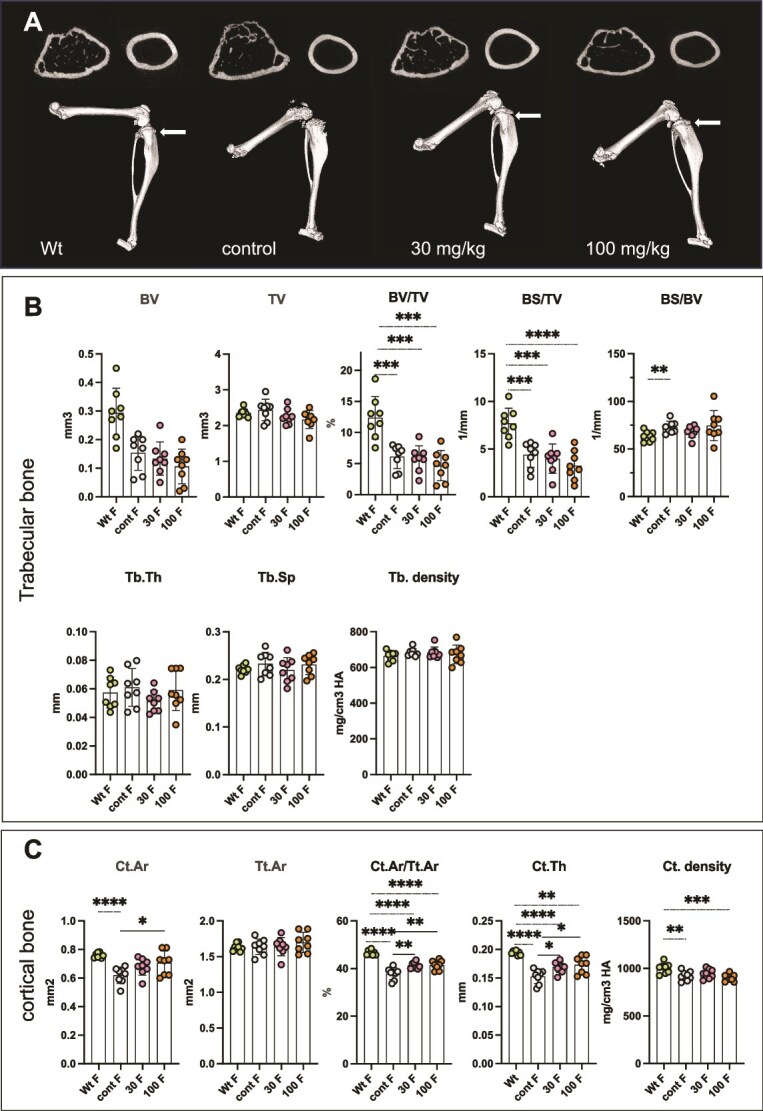
Micro-CT analysis on female mice. (A) 3D views of left hind limb. Top left: trabecular bone. Top right: cortical bone. White arrows indicate growth plate area. (B) Micro-CT analysis of trabecular bone. Parameters analyzed include bone volume (BV), tissue volume (TV), bone volume fraction (BV/TV), bone surface (BS), trabecular thickness (Tb.Th), trabecular separation (Tb.Sp), trabecular density (Tb. density), cortical area (Ct.Ar), tissue area (Tt.Ar), cortical thickness (Ct.Th), and cortical density (Ct.density). Wt: wild-type mice *Alpl^flox/flox^*, cont: *Alpl^Prx1/−^*, 30: *Alpl^Prx1/−^* mice with 30 mg/kg REV102, 100: *Alpl^Prx1/−^* mice with 100 mg/kg REV102 (*n* = 8).

**Figure 4:**
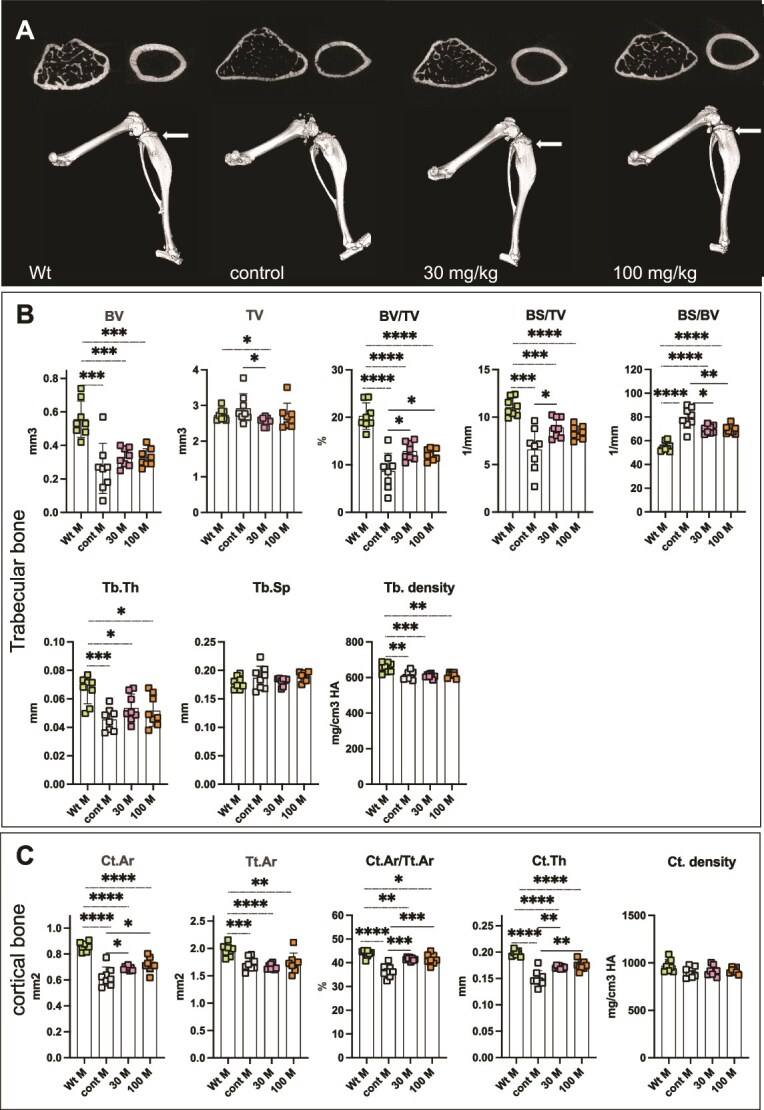
Micro-CT analysis on male mice. (A) 3D views of left hind limb. Top left: trabecular bone. Top right: cortical bone. (B) Micro-CT analysis of trabecular bone. Parameters analyzed include bone volume (BV), tissue volume (TV), bone volume fraction (BV/TV), bone surface (BS), trabecular thickness (Tb.Th), trabecular separation (Tb.Sp), trabecular density (Tb. density), cortical area (Ct.Ar), tissue area (Tt.Ar), cortical thickness (Ct.Th), and cortical density (Ct.density). Wt: wild-type mice *Alpl^flox/flox^*, cont: *Alpl^Prx1/−^*, 30: *Alpl^Prx1/−^* mice with 30 mg/kg REV102, 100: *Alpl^Prx1/−^* mice with 100 mg/kg REV102 (*n* = 8).

Three-dimensional images of hind limbs show abnormal or absent patella structure and the irregular shapes of the tibial epiphysis in *Alpl^Prx1/−^* control mice, but the patella structure was clearly recognized in REV102-treated mice (Figures 3A and 4A). The treated legs appear similar to the Wt legs, however, the growth plate area in the treated *Alpl^Prx1/−^* mice showed less mineralization than the growth plates of Wt mice (white arrows in Figures 3A and 4A). Micro-CT analysis revealed that bone volume (BV) in trabecular bones of female *Alpl^Prx1/−^* mice tended to have a wide range of variation and statistically significant effects by REV102 were not reached (Figure 3B); however, the cortical area per total area (Ct.Ar/Tt.Ar) and cortical thickness (Ct.Th) show statistically significant improvement (Figure 3C). In male *Alpl^Prx1/−^* mice, BV values were widely scattered in the untreated mice, however, in contrast to the female mice, BV values showed smaller standard deviation in the dosed *Alpl^Prx1/−^* male mice (Figure 4B). Bone volume fraction (BV/TV) indicates increased mineralization in the treated *Alpl^Prx1/−^* male mice, while the improvement did not reach the values seen in Wt mice (Figure 4B). Bone surface per bone volume (BS/BV) is higher in the *Alpl^Prx1/−^* mice than in Wt mice but lower in the treated *Alpl^Prx1/−^* male mice. Cortical area was also increased in the treated *Alpl^Prx1/−^* male mice and Ct.Ar/Tt.Ar and Ct.Th values indicate increased mineralization in the cortical bone of male *Alpl^Prx1/−^* mice with REV102 treatment, while these factors in treated animals did not match the levels of Wt mice (Figure 4C).

**Figure 5:**
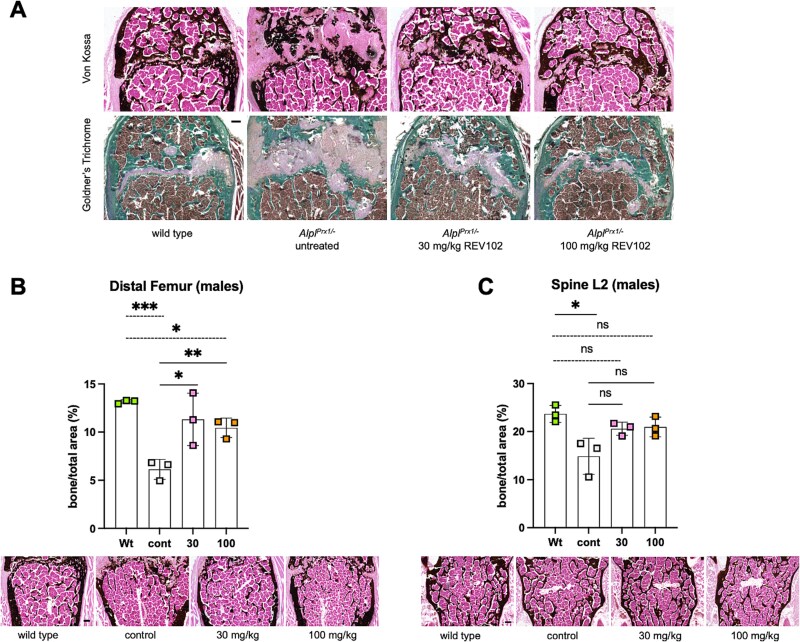
Bone morphometry analysis. (A) Growth plate views of distal femur from male mice stained with Von Kossa and Goldner’s trichrome stain. (B) Bone morphometry analysis on distal femur and representative sections. (C) Bone morphometry analysis on L2 vertebrate bone and representative sections. Three mice per cohort and at least three sections per mouse were used for the analysis. (*n* = 3). Black bars 200 μm.

*Alpl^Prx1/−^* mice show disorganized growth plates with thick layers of unmineralized zone, while *Alpl^Prx1/−^* mice treated with REV102 exhibited a reduced unmineralized zone, comparable to that of Wt mice (Figure 5A). Bone morphometry analysis on femurs indicated increased mineralization in the treated *Alpl^Prx1/−^* mice; however, the improvement in *Alpl^Prx1/−^* mice even with a 100 mg/kg dose did not reach the level of Wt mice (Figure 5B). In vertebrate bone L2 samples, a statistically non-significant small improvement was seen in the treated *Alpl^Prx1/−^* groups, but the ratio of the mineralized area approached similar values to those of Wt mice (Figure 5C). We did not observe any ectopic calcification in the metatarsophalangeal joints nor in the kidneys in either the 30 mg/kg or the 100 mg/kg treatment cohorts (Figures S5 and S6).

### Altered gene expression as a result of ENPP1 inhibition

**Figure 6:**
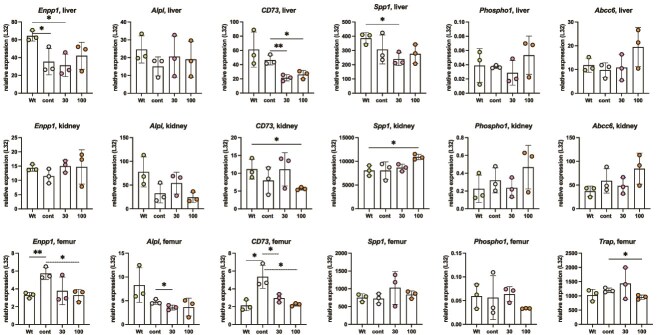
Quantitative RT-PCR analysis. Mouse ribosomal protein L32 was used for normalization. Biological replicates were three female mice, and technical replicates were done with quadruplets. Primers and gene IDs were shown in Table S1.

Liver, kidney, and bone are major organs that express both *Enpp1* and *Alpl*, therefore we looked for changes in the expression of these genes, as well as others associated with PP_i_ metabolism, in response to ENPP1 inhibition (Figure 6). *Alpl* expression by qRT-PCR was not significantly affected by the REV102 treatment in the three organs. *Enpp1* expression in the *Alpl^Prx1/−^* mice was low in the liver but high in bone, and ENPP1 inhibition reduced *Enpp1* expression in their bones. The signal of ENPP1 protein in the tibia bone was almost undetectable in Wt mice (Figure S7A); however, the bones from *Alpl^Prx1/−^* mice showed increased expression levels, which were reduced in the treated bones, in agreement with the qRT-PCR data showing upregulation of *Enpp1* in the *Alpl^Prx1/−^* mice. It was unexpected that ENPP1 is upregulated in the bone of *Alpl^Prx1/−^* mice, as plasma PP_i_ is higher than in Wt mice. *Enpp1* is downregulated in the liver of *Alpl^Prx1/−^* mice, while TNAP expression remains comparable to that in Wt mice.

Reduction of CD73 in the REV102-treated bones is also observed at the protein level (Figure S7B). The lower CD73 expression caused by REV102 treatment seen in the liver might be associated with reduced PP_i_ or AMP levels. The expression of either OPN (*Ssp1*) or *Phospho1* was not significantly changed except that the kidneys dosed with 100 mg/kg REV102 showed some upregulation. However, OPN signals detected in the kidneys were very low and no increase was observed by Western blot (Figure S7C). *Abcc6* did not exhibit significantly different expression, but *Alpl^Prx1/−^* mice treated with 100 mg/kg REV102 showed higher levels of *Abcc6* than other groups. *Abcc6* was not detectable in the femur by qRT-PCR using 1% of cDNA transcribed from 2 mg total RNA. *Trap* expression was not increased in the femurs of *Alpl^Prx1/−^* mice with 100 mg/kg REV102 indicating that bone resorption was not affected.

In the global *Alpl^−/−^* (*Alpl^tm1Jlm^*) mouse model of infantile HPP, plasma OPN was markedly elevated as phosphorylated OPN accumulated due to lack of TNAP,^32^ while in the current *Alpl^Prx1/−^* conditional knockout model, plasma OPN was not significantly elevated (Figure S8) in agreement with the qRT-PCR data (Figure 6). This result resembles the plasma OPN levels of a transgenic mouse over-expressing human TNAP under control of *Col1a1* promoter in the background of *Alpl^−/−^* exhibiting a milder bone phenotype,^33^ indicating that increase of plasma OPN may only be seen in the infantile model of HPP, which is in the fast-growing postnatal stages. In the case of male *Alpl^Prx1/−^* mice, plasma OPN was increased by REV102 treatment (Figure S8); however, we do not have information on the phosphorylation status or the organs that contributed to this increase. The tibial bones and kidneys were compared in Western blots and OPN was rather reduced in the treated females (Figure S7C) suggesting that the increased plasma OPN did not originate from the bone or the kidneys.

## Discussion

We previously reported that ENPP1 and TNAP are antagonistic regulators of bone mineralization, controlling local PP_i_ levels in the skeleton.^4^^,^^34^ ENPP1 is present in almost all tissues/cells at levels ranging from low to high in both humans and mice, including osteoblasts/chondrocytes, hepatocytes, proximal convoluted tubule cells in the kidneys, white adipocytes, testis, pancreas, skin, and the lens (https://www.proteinatlas.org, BioGPS http://biogps.org). In this first proof-of-concept study, we aimed at testing whether systemic administration of an ENPP1 inhibitor could lead to significant reductions in plasma PP_i_ concentrations that might be sufficient to improve skeletal mineralization in a mouse model of later-onset HPP. We purposely did not focus on testing this new therapeutic principle using the well characterized *Alpl^−/−^* model of infantile HPP,^35^^,^^36^ because homozygous pups die before weaning with severe seizures and apnea. This is due to accumulation in the circulation, but deficiency in the central nervous system,^37–39^ of another important physiological substrate of TNAP, pyridoxal 5′-phosphate, the most significant isoform of vitamin B6, and ENPP1 inhibition would not be expected to correct that crucial deficit. Indeed, the [*Enpp1^−/−^; Alpl^−/−^*] pups in our earlier studies,^4^^,^^34^ showed improved skeletal mineralization particularly in the axial skeleton, but the mice still died before weaning, due to the persistent dysfunction in vitamin B6 metabolism in the double knockout pups. Recent studies confirmed those findings and documented rescue of the skull phenotype in [*Enpp1^−/−^; Alpl^−/−^*] pups with marked reduction in the incidence of craniosynostosis, particularly of the coronal fusion, a prominent feature observed in up to 40% of the *Alpl^−/−^* mice.^40^^,^^41^ The reason for the differential outcome in that double genetic experiment demonstrating rescue of the axial skeletal phenotype but persistent osteomalacia in the appendicular skeleton, is attributed to the higher levels of *Enpp1* mRNA expression in the axial skeleton^42^ with comparable levels of expression of ENPP1 and TNAP protein in calvaria, but not in the femurs/tibias.^43^ Indeed, our recent data using proteoliposomes harboring ENPP1 and TNAP in different ratios demonstrated that the pyrophosphohydrolase function of ENPP1 and the phosphohydrolase activity of TNAP act synergistically to produce a P_i_/PP_i_ ratio conducive to mineralization and the synergism is maximal when the two enzymes are present at equimolar concentrations.^44^ With that background in mind, we purposefully decided to use the *Alpl^Prx1/−^* mice, which display pronounced osteomalacia in the appendicular skeleton but not in the axial skeleton, as a purposefully difficult model to test the efficacy of ENPP1 inhibition.

We administered REV102 via diet instead of gavage since a long dosing period was necessary to observe changes in bone phenotype. Daily gavage for over 100 d would be highly stressful to the animals. We also aimed at minimizing disturbing the animals during daytime when mice sleep and bone growth likely occurs, as bone metabolism is regulated by the circadian rhythm. We performed multiple pilot PK/PD studies spanning 1-10 d period using diets containing several doses of REV102 to compare the reduction of plasma PP_i_ and drug levels. The PP_i_ reduction obtained after the 105-d dosing was as expected (Figure 1I), but, notably, females showed a lesser negative correlation between drug and PP_i_ levels in both doses (Figure 1D-G). We also observed that females (Wt and *Alpl^Prx1/−^* mice) show higher levels of both plasma AP and plasma PP_i_ than males (Wt and *Alpl^Prx1/−^* mice), as shown in Figure 1H and I. We have seen similar sex-related trends in plasma AP and PP_i_ in unrelated studies as well, implying that an unknown mechanism that influences plasma AP and PP_i_ levels exists in females. Figure 1H shows untreated control female *Alpl^Prx1/−^* mice had higher plasma AP than treated females, while plasma AP levels were unchanged between untreated and treated males. We surmise the elevated AP levels in the female mutant mice are due to unrelated factors such as a cage effect, as estrus synchronization occurs in a cage. A particular female mouse, ID 628, gave 1.011 μM plasma PP_i_ despite of the high drug level (770 ng/mL) (Figure 1F), on the other hand 628 shows improved mineralization in X-ray analysis (Figure S2). It is possible that the increased PP_i_ in the mouse 628 at collection time was a transient phenomenon. Plasma PP_i_ levels in untreated mice (Wt and *Alpl^Prx1/−^*) show wide variations, while values from REV102-treated animals were well clustered (Figure 1I), indicating that REV102 overrides other mechanisms that influence plasma PP_i_ levels.

Body weight of treated *Alpl^Prx1/−^* females became close to Wt females, but male *Alpl^Prx1/−^* mice did not gain weight as much as Wt males (Figure 1C) likely because the male mutant mice exhibit more severe bone phenotype than female *Alpl^Prx1/−^* mice. The body weight difference between male Wt and *Alpl^Prx1/−^* mice is larger than in females (Figure 1C) and X-ray analysis showed that impaired mineralization in the epiphysis of male *Alpl^Prx1/−^* mice was more prominent than in female mutants (Figure 2A), and femur length of male *Alpl^Prx1/−^* mice was even shorter than those of female mutant mice (average 13.73 and 13.34 mm, respectively). We do not know why the distal femur of male mutant mice is more affected, but we surmise that the load on the femur is greater in males due to the heavier body weight. Appearance of the patella bone and shape of the epiphyses of treated mice were similarly improved in the X-ray views, and the length of femur and tibia became longer in both genders. However, micro-CT analysis reveals that significant improvement in trabecular bone is only significant in male mice (Figures 3B and 4B). The wide variation in BV values remaining among the treated females may suggest female *Alpl^Prx1/−^* mice are more resistant to the treatment. In the cortical bones, both female and male mutants showed increased mineralization (Figures 3C and 4C). It is expected that the ENPP1 inhibitor reaches the bone marrow cavity through the invading blood vessels along with mesenchymal stem cells to be differentiated into osteoblasts that mineralize the surface of the trabecular bones. REV102 dosing was started at 25 dpn, which is after formation of the secondary ossification centers, and it is possible that dosing longer than 105 d could have shown better improvement in the trabecular bones. Another possibility is that higher PP_i_ levels throughout the mutants’ embryogenesis and postnatal stages already affected bone formation making thinner/fewer trabecular bones, so that treatment starting at dpn 25 did not overcome the deficiency. It is also reasonable to anticipate that improved ENPP1 inhibitor compounds might exhibit superior improvement in the bone phenotype than we observed here. There are a myriad of commercial ENPP1 inhibitors reported in the literature and more being development by pharmaceutical companies, primarily for use in tumor immunology. Many of those compounds could potentially also be tested for improved efficacy.

*Enpp1* knockout mice exhibit a tiptoe walking phenotype due to stiffening of the joints with ectopic calcification.^4^ We have never observed such tiptoe walking behavior in the mice treated with REV102, and X-ray images of the metatarsophalangeal joints from treated mice show no ectopic calcification (Figure S5). Furthermore, *Enpp1* knockout mice, in which plasma PP_i_ levels are almost unmeasurable, do not show ectopic calcification in the kidney when fed a regular chow and only exhibit kidney calcification when they consume a high phosphate–low magnesium diet.^45^ Our base diet in the current study does not have high phosphate nor low magnesium. We examined the kidneys of the treated mice and, as expected, found no ectopic mineral deposition (Figure S6).

A limitation of our study is that we have confined ourselves to a proof-of-concept evaluation of the ability of ENPP1 to reduce PP_i_ in vivo, assessing the consequences of those changes on bone mineralization using our *Alpl^Prx1/−^* model of later-onset HPP. Given that another known function of ENPP1 is the hydrolysis of cyclic guanosine monophosphate-adenosine monophosphate (cGAMP) that activates the Stimulator of Interferon Genes (STING) pathway,^46^^,^^47^ future studies should focus on assessing any deleterious or beneficial effects of modulating the STING pathway in the context of HPP. This is relevant because both ENPP1 and TNAP are members of the larger AP superfamily of enzymes that display considerable structural similarity,^48^ some redundant functions in phosphate generation, and both are also involved in immunomodulation, with ENPP1 acting mainly as pro-inflammatory and TNAP as anti-inflammatory.^47^^,^^49^ Therefore, future studies should evaluate the consequences of ENPP1 inhibition in the context of HPP, investigating their role in immune regulation. At present, the mice consuming 30 and 100 mg/kg REV102 diet showed normal growth (Figure 1A-C) and a comprehensive plasma analysis found no abnormality in the treated animals (Figure S1), indicating that use of REV102 at these doses is nontoxic and has potential to be used in humans. The presence of calcium pyrophosphate dihydrate (CPPD) has also been described in patients with HPP,^8^ and we see a potential for using ENPP1 inhibitors to reduce that burden. However, whether that indication would be best addressed with systemic or local injections would need to be evaluated in clinical trials, as we have not observed the presence of CPPD in the *Alpl^Prx1/−^* mouse model.

To conclude, in this study, we have shown that oral dosing of an ENPP1 inhibitor, REV102, markedly reduced plasma PP_i_ levels and enhanced bone mineralization in the *Alpl^Prx1/−^* mouse model of later-onset HPP without any noticeable side effects. Our data indicate that ENPP1 inhibitors have the potential to be used as therapeutic drugs to improve the bone phenotype in HPP patients.

## Supplementary Material

Suppl_Fig_1_zjaf136

Suppl_Fig_2_zjaf136

Suppl_Fig_3_zjaf136

Suppl_Fig_4_zjaf136

Suppl_Fig_5_zjaf136

Suppl_Fig_6_zjaf136

Suppl_Fig_7_zjaf136

Suppl_Fig_8_zjaf136

Suppl_Table_zjaf136

Legends_to_the_supplemental_table_and_figures_zjaf136

## Data Availability

The data that supports the findings of this study are presented in the printed version and in the supplementary material of this article. Additional data related to this study might be available upon request to the authors.
