# Supplementary figures and images for: ENPP1 inhibition as a therapeutic approach for later-onset hypophosphatasia
Source: J Bone Miner Res. 2025 Oct 6;41(3):310–23. doi: 10.1093/jbmr/zjaf136 (PMC13017400; doi:10.1093/jbmr/zjaf136)

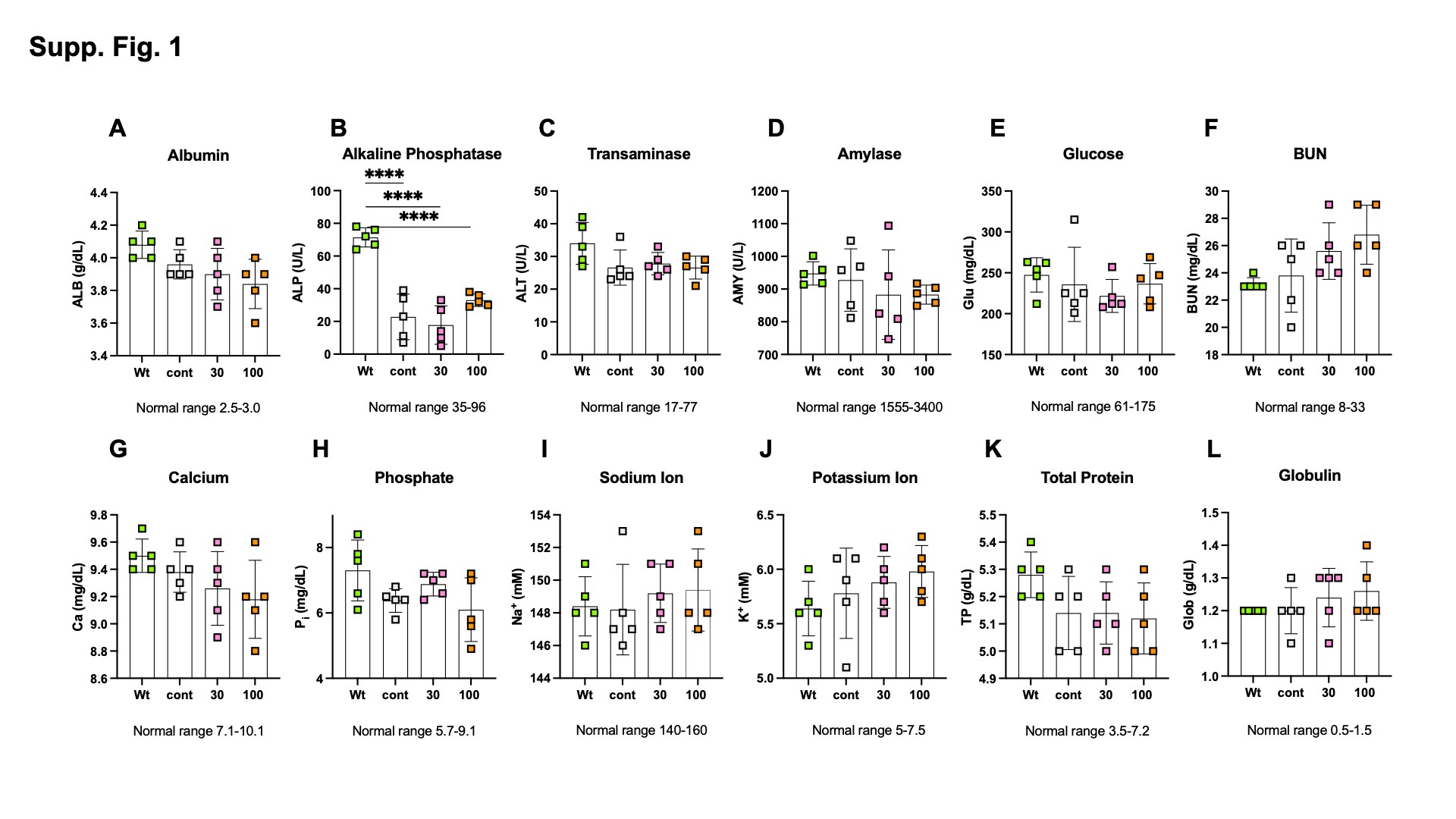

Supplement: Suppl_Fig_1_zjaf136 [file suppl_fig_1_zjaf136.jpeg]

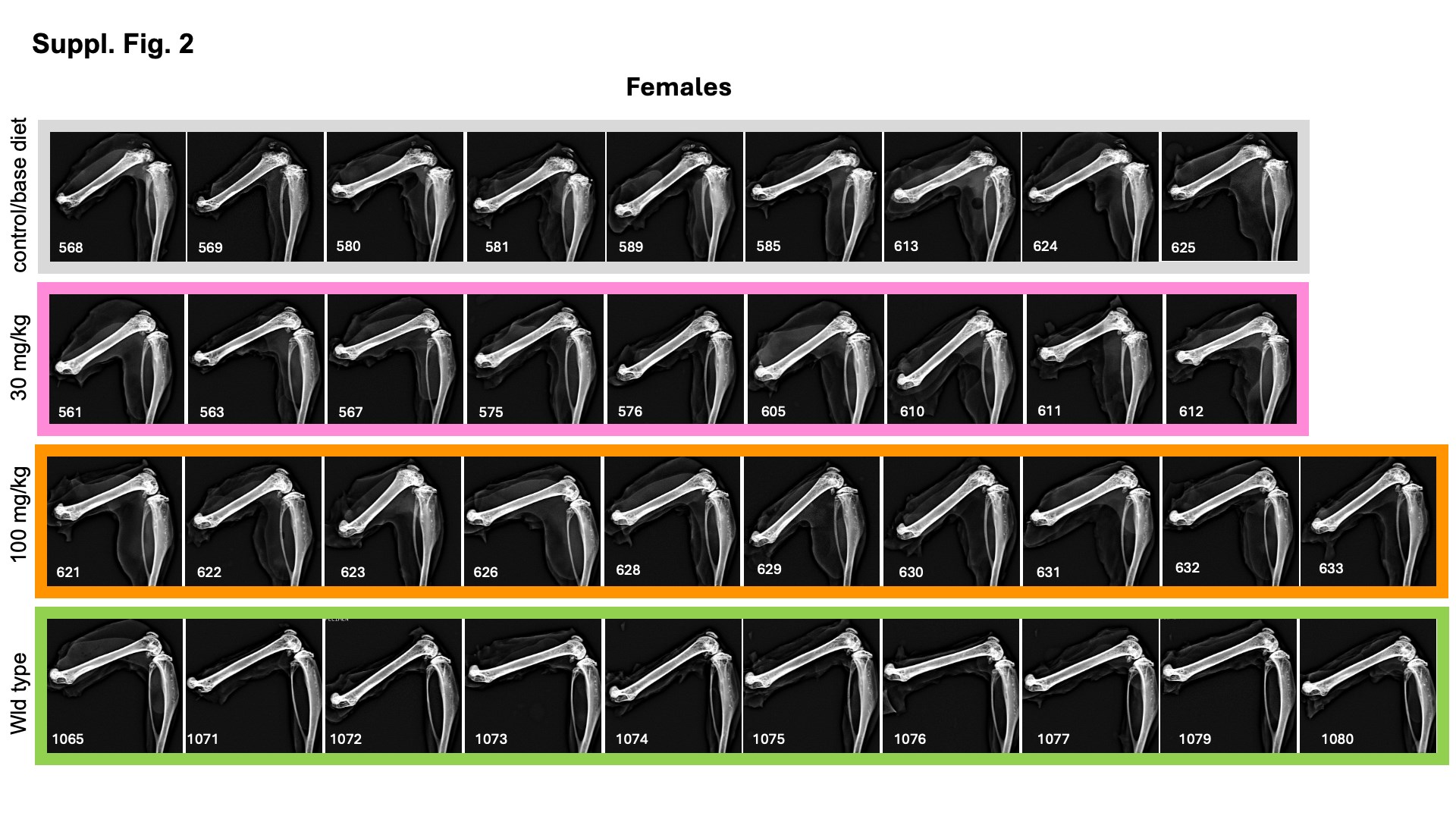

Supplement: Suppl_Fig_2_zjaf136 [file suppl_fig_2_zjaf136.jpeg]

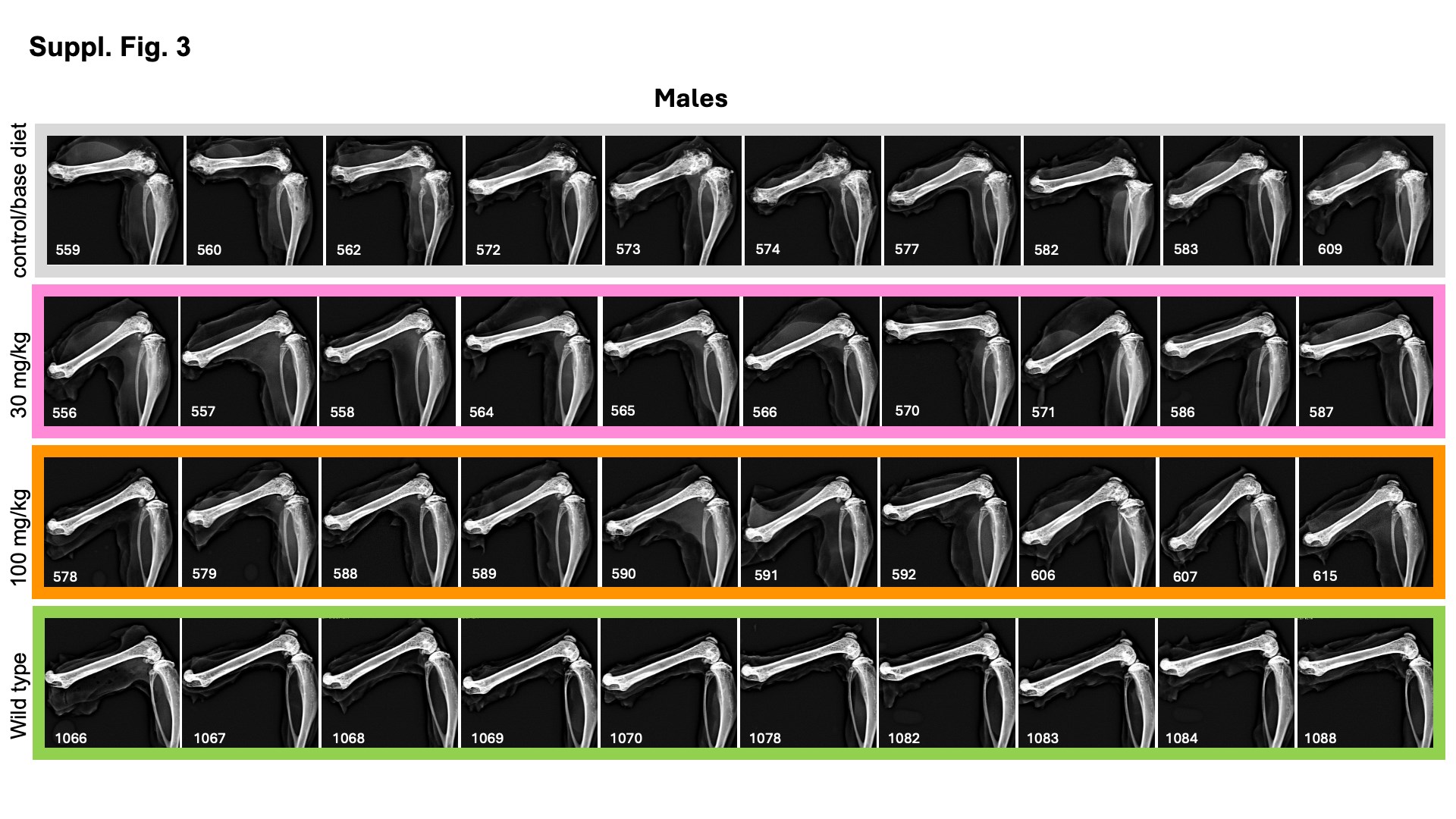

Supplement: Suppl_Fig_3_zjaf136 [file suppl_fig_3_zjaf136.jpeg]

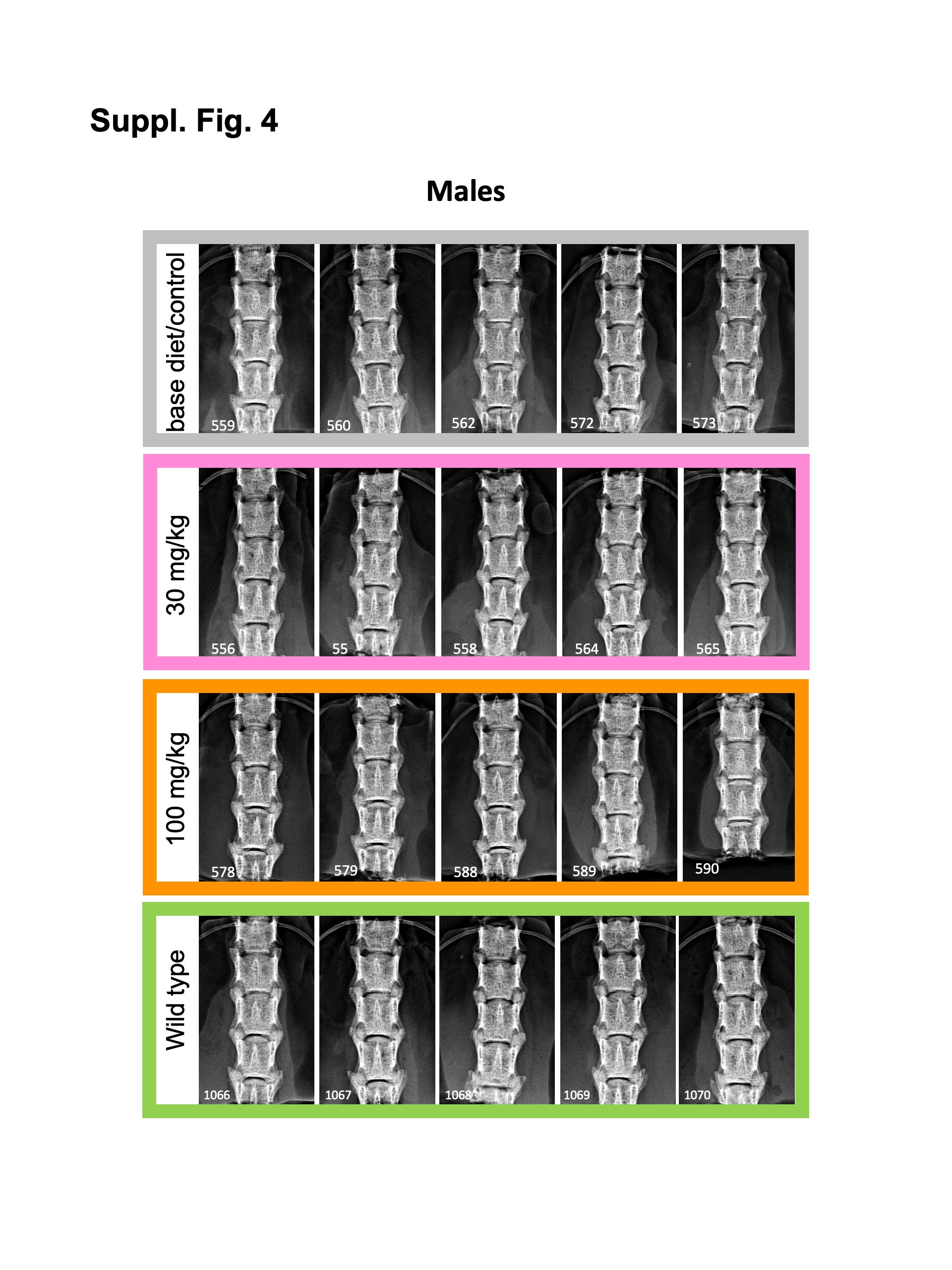

Supplement: Suppl_Fig_4_zjaf136 [file suppl_fig_4_zjaf136.jpeg]

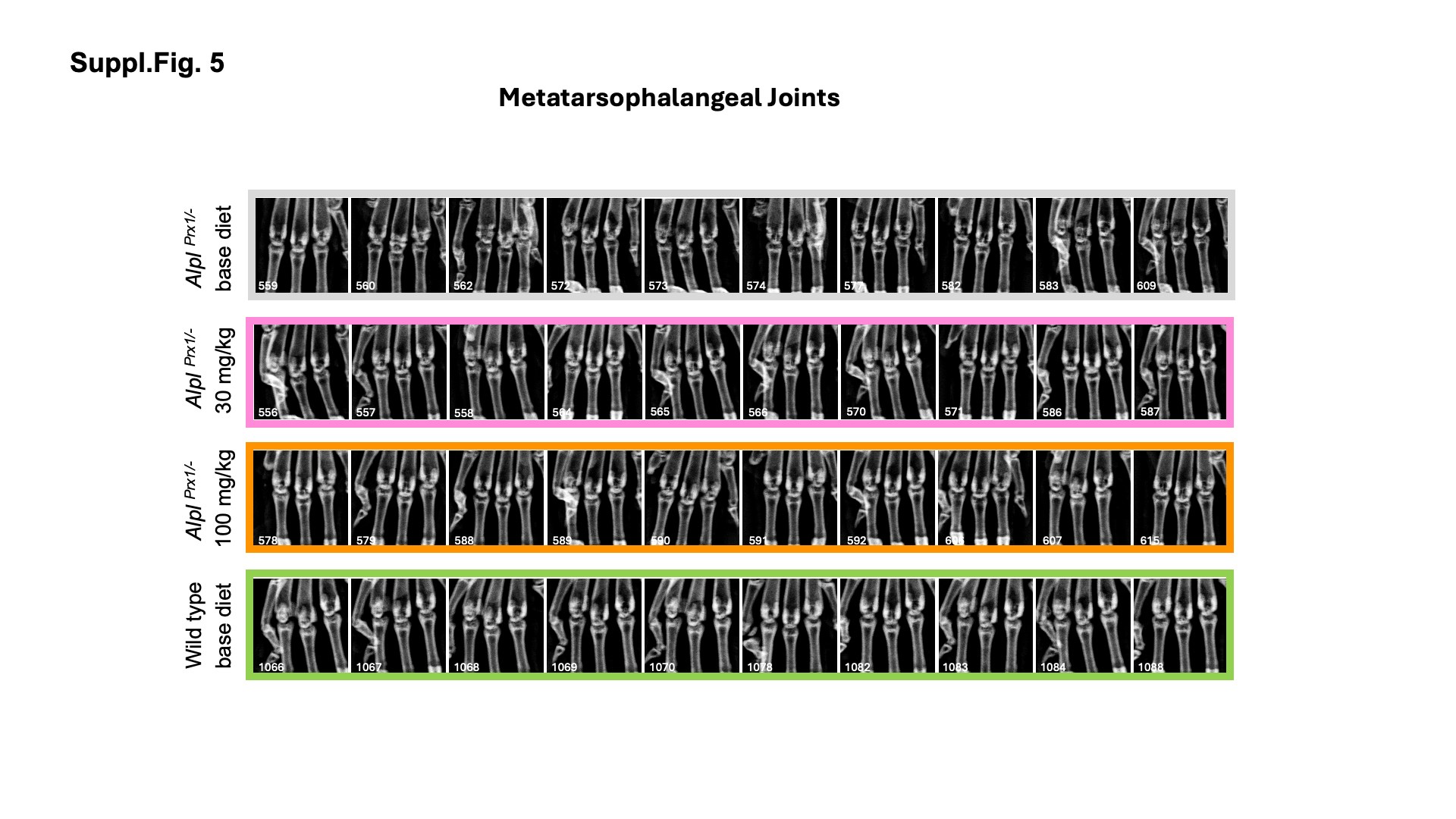

Supplement: Suppl_Fig_5_zjaf136 [file suppl_fig_5_zjaf136.jpeg]

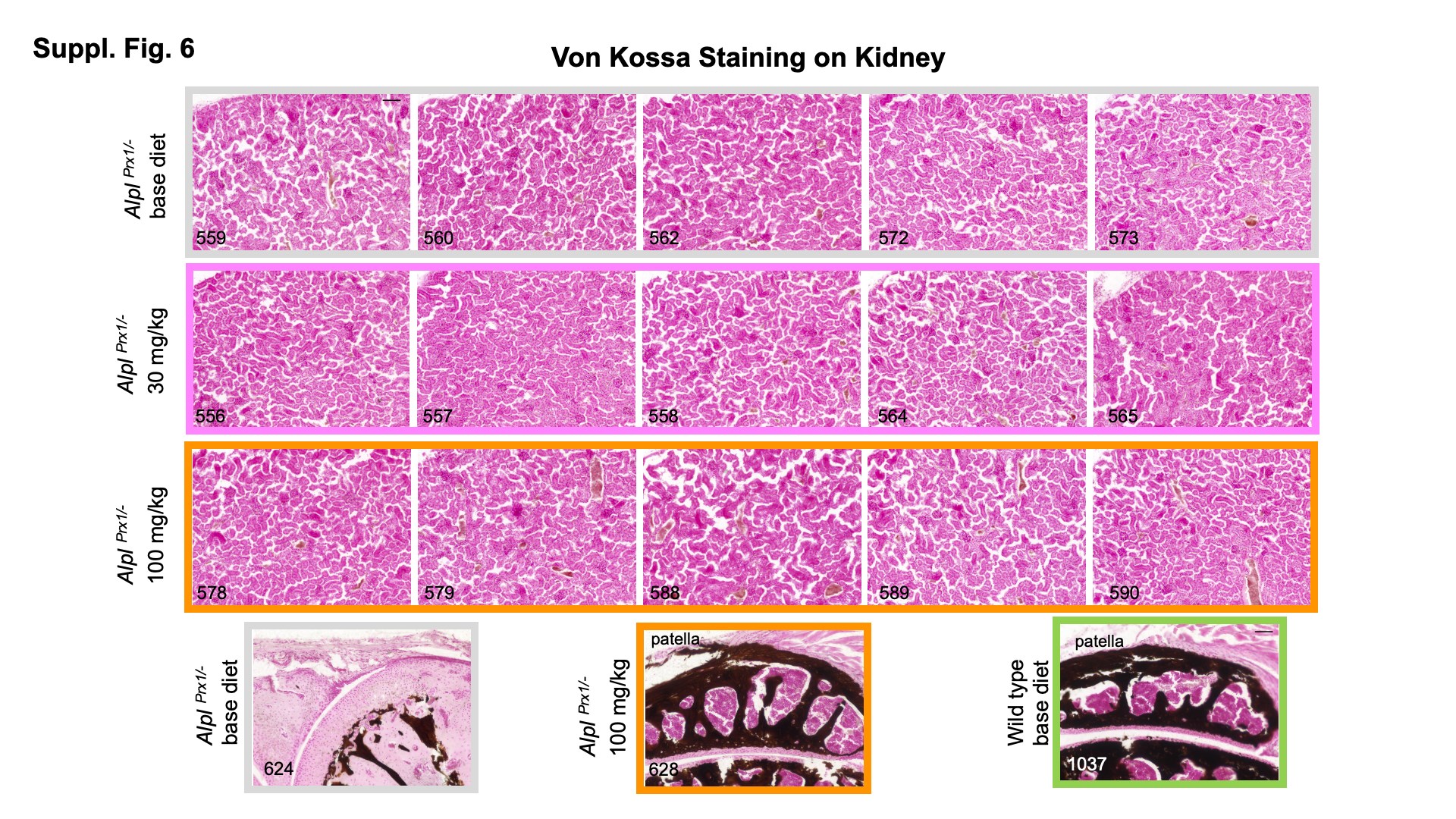

Supplement: Suppl_Fig_6_zjaf136 [file suppl_fig_6_zjaf136.jpeg]

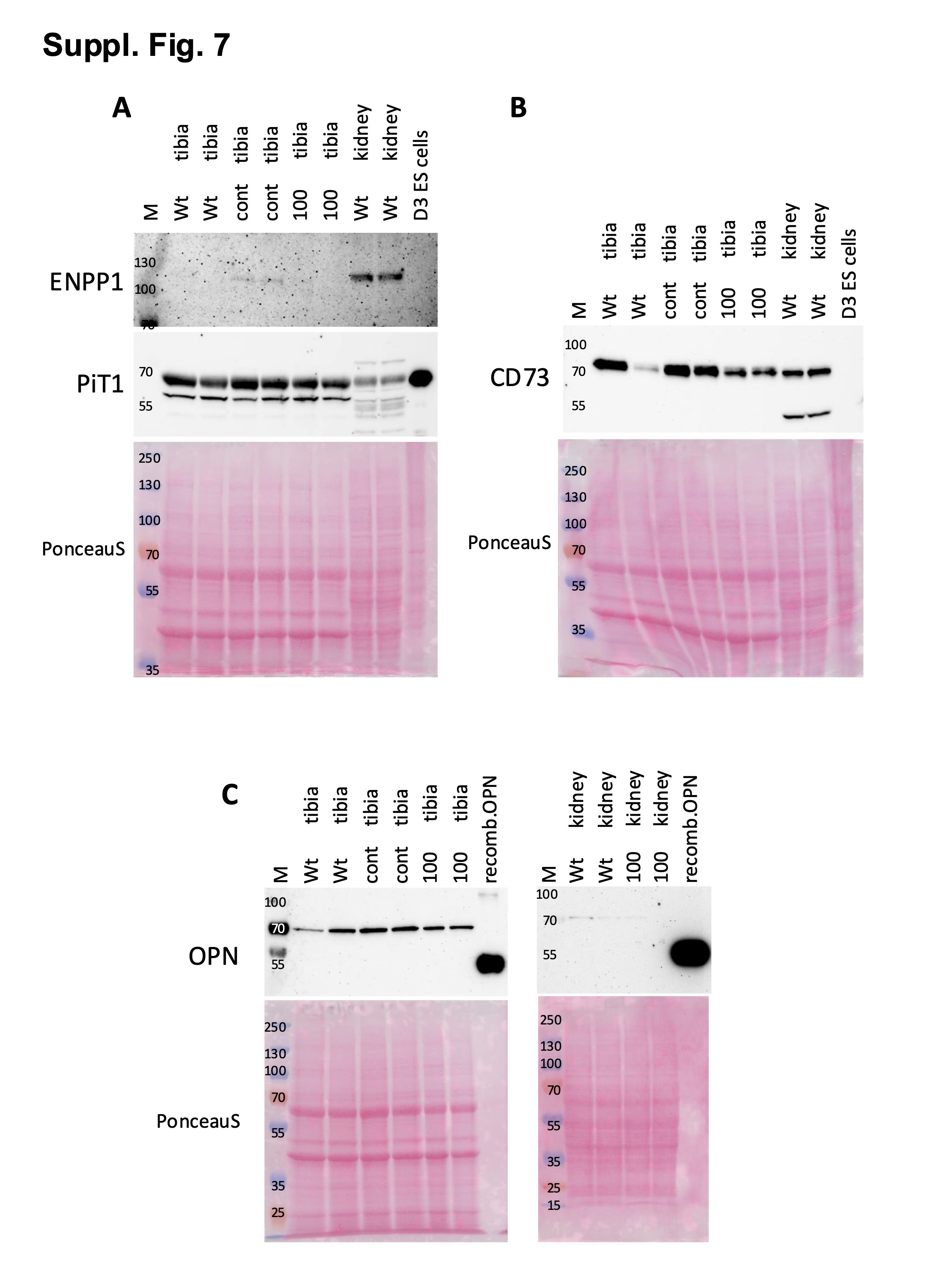

Supplement: Suppl_Fig_7_zjaf136 [file suppl_fig_7_zjaf136.jpeg]

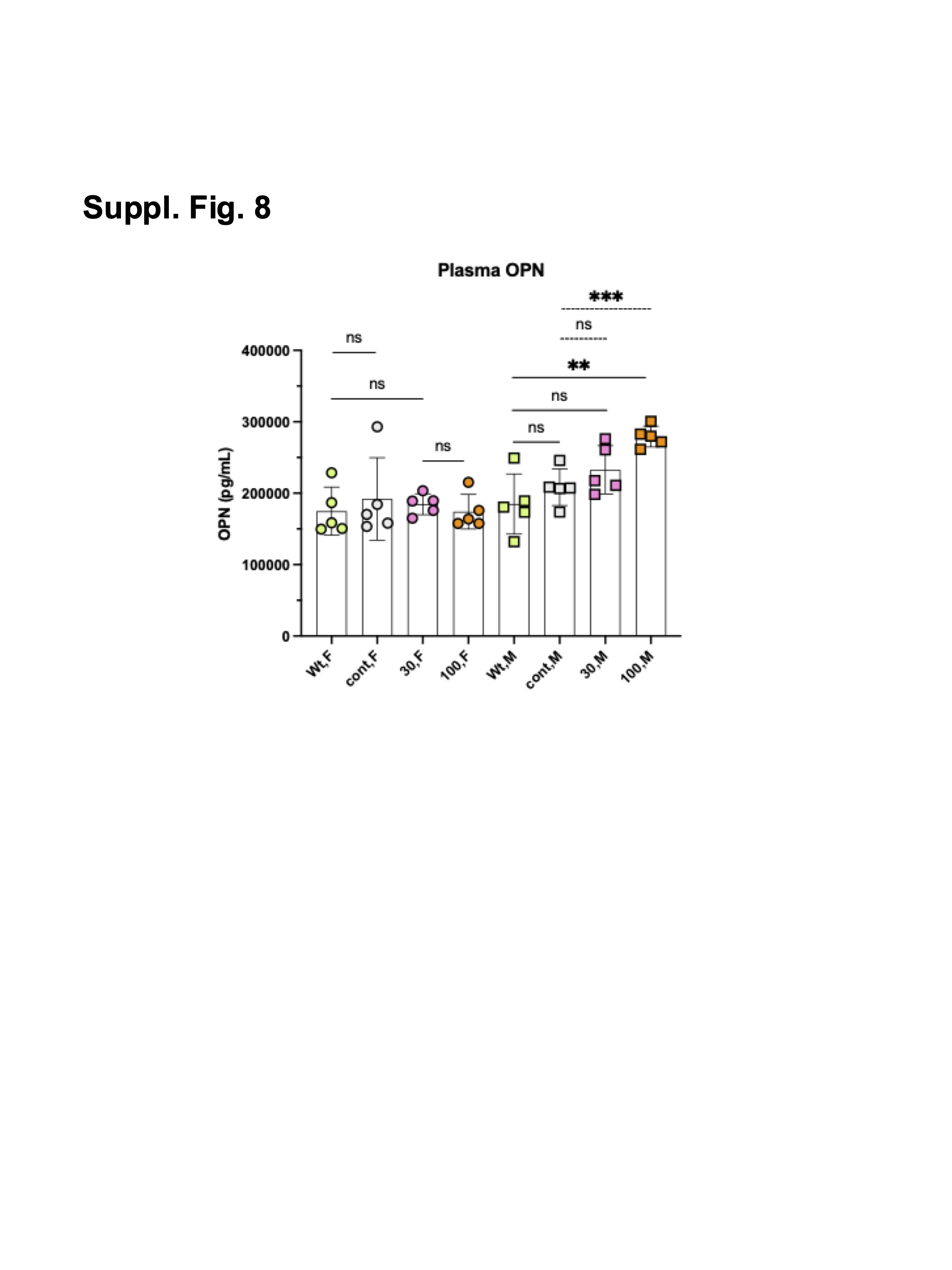

Supplement: Suppl_Fig_8_zjaf136 [file suppl_fig_8_zjaf136.jpeg]

**Supplemental Table**


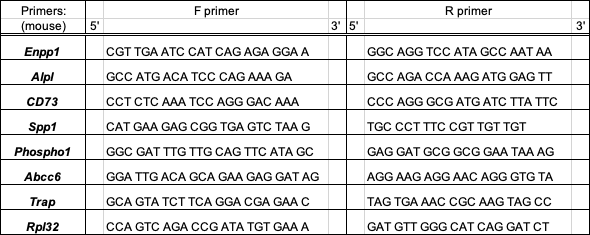

Supplement: Suppl_Table_zjaf136 [file suppl_table_zjaf136.docx]
