# Supplementary material for: ENPP1 inhibition as a therapeutic approach for later-onset hypophosphatasia
Source: J Bone Miner Res. 2025 Oct 6;41(3):310–23. doi: 10.1093/jbmr/zjaf136 (PMC13017400; doi:10.1093/jbmr/zjaf136)
Supplement: Legends_to_the_supplemental_table_and_figures_zjaf136 [file legends_to_the_supplemental_table_and_figures_zjaf136.docx]

**Legends to the supplemental table and figures**

Supplemental Table: Sequences of the primers and gene IDs used in qRTPCR.

Supplemental Fig. 1: Plasma factors measured by Abaxis VetScan2 (n=5, males). Albumin (A), alkaline phosphatase (B), transaminase (C), amylase (D), glucose (E), BUN (F), Calcium (G), phosphate (H), sodium ion (I), potassium ion (J), total protein (K) and globulin (L). All animals showed 0.3 mg/dL total bilirubin and low creatinine (<0.2). Only significant difference was observed in alkaline phosphatase between wild type and the *Alpl^Prx1/-^* mice. Other values did not show significant changes (not indicated in graphs). Albumin and glucose in this mouse background were higher than normal range (A, E), while amylase was lower than normal range (D).

Supplemental Fig. 2: X-ray images of the left hind limb (femur and tibia/fibula) from all the female mice obtained. Numbers at the bottom left corner in each picture are mouse ID numbers.

Supplemental Fig. 3: X-ray images of the left hind limb (femur and tibia/fibula) from all the male mice obtained. Numbers at the bottom left corner in each picture are mouse ID numbers.

Supplemental Fig. 4: X-ray images of the spine from five male mice. Numbers at the bottom left corner in each picture are mouse ID numbers.

Supplemental Fig. 5: X-ray images of the metatarsophalangeal joints from male mice in the control, 30 mg/kg dose and 100 mg/kg dose groups and wild type mice. Numbers at the bottom left corner in each picture are the mouse ID numbers.

Supplemental Fig. 6: Von Kossa staining of kidney sections from male mice in the control, 30 mg/kg dose, and 100 mg/kg dose groups. Technical positive control samples are knee joints from untreated *Alpl^Prx1/-^*, *Alpl^Prx1/-^*  treated with 100 mg/kg REV102, and a wild type mouse. Patella in the treated *Alpl^Prx1/-^*  mouse is well mineralized. Numbers at the bottom left corner in each picture are mouse ID numbers. All images are the same magnification: Bar 100 µm.

Supplemental Fig. 7: Plasma OPN levels were measured by ELISA. (n=5, females and males)

Supplemental Fig. 8: Western blot for ENPP1 and P_i_T1 (A), CD73 (B) and OPN (C). All the samples applied are 100 mg protein except that the recombinant OPN is 30 ng. Ponceau S staining of each membrane indicates the amount of total protein in each lane.
